# Supplementary material for: Lighting in the Home and Health: A Systematic Review
Source: Int J Environ Res Public Health. 2021 Jan 12;18(2):609. doi: 10.3390/ijerph18020609 (PMC7828303; doi:10.3390/ijerph18020609)
Supplement: Supplementary file 1 [file ijerph-18-00609-s001.pdf]

Lighting in the home and health: a systematic review

Oluwapelumi Osibona, Bethlehem D Solomon and Daniela Fecht

Content

Table S1. Search strategy for lighting in the home and health **Page 1**

Table S2. Quality assessment score using the Newcastle-Ottawa Scale **Page 4**

Table S1. Search strategy for lighting in the home and health

1.1 MEDLINE Search strategy

	Search Term
1	Lighting/
2	sunlight.mp. or Sunlight/
3	nightlight*.mp.
4	daylight*.mp.
5	bulb*.mp.
6	lighting.mp.
7	natural light*.mp.
8	artificial light*.mp.
9	lighting*1.mp.
10	illumination.mp.
11	Light/
12	residential light*.mp.
13	domestic light*.mp.
14	artificial light/ or environmental control/ or illumination/ or lamps/ or light transmission/ or visibility/
15	lamp*1.mp.
16	1 or 2 or 3 or 4 or 5 or 6 or 7 or 8 or 9 or 10 or 11 or 12 or 13 or 14 or 15
17	housing.mp. or Housing/ or Public Housing/ or Housing for the Elderly/
18	(hous* or home*1 or resident* or apartment*).mp.
19	17 or 18
20	16 and 19
21	exp animals/ not humans.sh.
22	20 not 21
23	health.mp. or exp Health/ or exp Adolescent Health/
24	22 and 23
25	Mental Health/ or mental health.mp.
26	injur*.mp.
27	accidental falls/ or exp accidents, home/
28	fall*.mp.
29	23 or 25 or 26 or 27 or 28
30	22 and 29
31	streetlight*.mp.
32	16 or 31
33	19 and 32
34	33 not 21

35	23 and 34
36	29 and 34
37	Public Health/
38	29 or 37
39	34 and 38
40	public health/ or exp epidemiology/ or exp preventive medicine/
41	38 or 40
42	34 and 41
43	exp Public Health/
44	38 or 43
45	34 and 44

1.2 Embase Search Strategy:

	Search Term
1	lighting.mp. or illumination/
2	sunlight/ or sunlight.mp.
3	nightlight*.mp.
4	daylight*.mp.
5	bulb*.mp.
6	natural light*.mp.
7	artificial light*.mp.
8	lighting*1.mp.
9	illumination.mp.
10	light/
11	residential light*.mp.
12	domestic light*.mp.
13	artificial light/ or environmental control/ or illumination/ or lamps/ or light transmission/ or visibility.mp.
14	lamp*1.mp.
15	streetlight*.mp.
16	1 or 2 or 3 or 4 or 5 or 6 or 7 or 8 or 9 or 10 or 11 or 12 or 13 or 14 or 15
17	housing.mp. or Housing/ or Public Housing/ or Housing for the Elderly/
18	(hous* or home*1 or resident* or apartment*).mp.
19	17 or 18
20	16 and 19
21	(exp animals/ or nonhumans/) not exp human/
22	20 not 21
23	exp child health/ or exp health/ or health.mp. or exp adolescent health/
24	22 and 23
25	mental health.mp. or mental health/
26	injury/ or injur*.mp.
27	exp falling/
28	fall*.mp.
29	23 or 25 or 26 or 27 or 28
30	22 and 29
31	exp public health/

32	29 or 31
33	22 and 32

1.3 SCOPUS search strategy

Search Term
lighting AND (home OR housing) AND (health OR public health)

Table S2. Quality assessment score using the Newcastle-Ottawa Scale

Author, Year	Selection	Comparability	Outcomes	Total score
Case-control studies				
Rahayu, 2015[28]	★★	★	★★	5
Chen, 2017[33]	★★	★	★★	5
Woldesemayat, 2014[36]	★★	NA	★★	4
Savitha, 2007[37]	★★★	NA	★★	5
Mashreky, 2010[38]	★★★	NA	★★	5
Camilloni, 2011[39]	★★	★★	★	5
Isberner, 1998[41]	★★	NA	★★	4
O'Leary, 2006[45]	★★	★★	★	5
Cohort studies				
Obayashi, 2018[52]	★★★	★★	★★	7
Obayashi, 2014[54]	★★	★★	★★★	7
Cross-sectional studies				
Ichimori, 2013[31]	★★★	NA	★★	5
Kumar, 2001[29]	★★	NA	★★	4
Brown, 2011[30]	★★	★★	★★	6
Youngstedt, 2004[32]	★★★	NA	★★	5
Patel, 2019[27]	★★	★★	★★	6
Shi, 2014[40]	★★★	★★	★★	7
Czepita, 2004[42]	★★	NA	★★★	5
Kayaba, 2014[44]	★★	★★	★★	6
Obayashi, 2015[46]	★★★★	★★	★★★	9
Obayashi, 2013[47]	★★★	★★	★★★	8
Obayashi, 2014[48]	★★★	★★	★★★	8
Obayashi, 2014[49]	★★★★	★★	★★★	9
Obayashi, 2013[51]	★★★★	★★	★★	8
Obayashi, 2014[53]	★★★★	★★	★★★	9
Intervention studies				
Brunnstrom, 2004[34]	★★	★	★★	5
Falkenberg, 2019[35]	★★★	★★	★★	7
Hopkins, 2017[43]	★★	★★	★★	6
Yamauchi, 2014[50]	★★★	NA	★★★	6

Abbreviations: NA – not applicable
